# Age- and Sex-Related Outcomes in Patients with Sepsis or Septic Shock: A Prospective Monocentric Cohort Study

**DOI:** 10.3390/jcm15114203

**Published:** 2026-05-29

**Authors:** Omar Hajji, Michael Behnes, Jonas Rusnak, Schanas Jawhar, Lea Marie Brück, Floriana Dulatahu, Svetlana Hetjens, Mohammad Abumayyaleh, Ibrahim Akin, Tobias Schupp, Kathrin Weidner

**Affiliations:** 1 Department of Cardiology, Haemostaseology and Medical Intensive Care, University Medical Centre Mannheim, Medical Faculty Mannheim, Heidelberg University, 68167 Mannheim, Germany; omarhajji07@gmail.com (O.H.); michael.behnes@umm.de (M.B.); schanas.jawhar@umm.de (S.J.); marie.brueck@web.de (L.M.B.); floriana.rauschenberg@umm.de (F.D.); mohammad.abumayyaleh@umm.de (M.A.); ibrahim.akin@umm.de (I.A.); kathrin.weidner@umm.de (K.W.); 2 Department of Cardiology, Angiology and Pneumology, University Hospital Heidelberg, 69120 Heidelberg, Germany; jonas.rusnak@med.uni-heidelberg.de; 3 Department for Biostatistics, Medical Faculty Mannheim, Heidelberg University, 60590 Mannheim, Germany; svetlana.hetjens@medma.uni-heidelberg.de

**Keywords:** sex, age, sepsis, septic shock, ICU, mortality

## Abstract

**Background**: Despite advances in the diagnosis and management of sepsis and septic shock (SS), sepsis is still characterized by mortality rates of up to 30% at 30 days, underlining the need for optimized risk stratification. This study investigated the influence of age and sex on short-term mortality in patients with sepsis and SS. **Methods**: This prospective, single-center, observational study included consecutive patients with sepsis/SS treated at a medical intensive care unit (ICU) of a University Medical Center between 2019 and 2021. Sepsis and SS were defined according to Sepsis-3 criteria. Patients were stratified by sex and age (≤75 vs. >75 years and per one-year increase). The primary endpoint was 30-day all-cause mortality. Survival was assessed by Kaplan–Meier and multivariable Cox regression analyses. **Results**: A total of 361 patients were included after screening 2596 ICU patients. Baseline characteristics were largely similar between men and women, whereas older patients had a greater burden of cardiovascular comorbidities and higher illness severity; 30-day mortality did not differ between men and women (51.9% vs. 52.3%; *p* = 0.948), whereas patients aged >75 years had higher 30-day mortality than younger patients (60.2% vs. 47.9%; *p* = 0.027). In multivariable Cox regression, age > 75 years remained independently associated with mortality (HR 1.391, 95% CI 1.021–1.896; *p* = 0.036), while sex was not (HR 1.016, 95% CI 0.740–1.396; *p* = 0.920). Sensitivity analysis using age as a continuous variable confirmed this association. **Conclusions**: In this prospective, registry-based, ICU cohort, older age was independently associated with higher short-term mortality in patients with sepsis or SS, whereas sex was not. Sex-related differences in treatment intensity and organ dysfunction markers were nevertheless observed and warrant further investigation.

## 1. Introduction

Sepsis is defined as life-threatening organ dysfunction caused by a dysregulated host response to infection, whereas septic shock (SS) represents a more severe subset characterized by profound circulatory, cellular, and metabolic abnormalities associated with a substantially increased risk of death [1,2,3]. Despite advances in recognition and management, sepsis remains a major global health challenge, accounting for an estimated 48.9 million incident cases and 11.0 million sepsis-related deaths worldwide in 2017. Current international guidelines emphasize early identification and timely, evidence-based treatment, but outcomes remain highly heterogeneous across patient subgroups [4,5]. Recognition of sepsis as a medical emergency is fundamental to its management. This premise prompted the introduction of the “1-h bundle” in the revised Surviving Sepsis Campaign (SSC) guidelines, consisting of lactate measurement, blood culture collection before antibiotic administration, prompt broad-spectrum antibiotic therapy, adequate fluid resuscitation, and vasopressor support when indicated. These recommendations reflect the explicit emphasis on the immediate initiation of resuscitation and management [5,6,7].

Besides recognized risk factors like age, immunosuppressive diseases, and chronic conditions such as diabetes and chronic obstructive pulmonary disease (COPD), sex may also influence sepsis outcomes [3,8]. Age is one of the most important determinants of susceptibility to sepsis and prognosis after its onset. Older adults are disproportionately affected because aging is accompanied by immunosenescence, multimorbidity, frailty, reduced physiological reserve, and more atypical clinical presentations, all of which may complicate diagnosis and limit recovery [9,10]. In intensive care unit (ICU) cohorts, very old patients with sepsis or SS have been reported to experience slightly higher mortality than younger elderly patients, underscoring the importance of age-stratified analyses rather than treating sepsis as a uniform syndrome across adulthood [11]. 

Sex is another potentially important but incompletely understood source of heterogeneity in sepsis. Experimental and clinical data suggest that sex hormones, sex-chromosome-related immune regulation, comorbidity patterns, and differences in healthcare use may all influence susceptibility, organ dysfunction, treatment intensity, and recovery [12,13]. However, evidence remains inconsistent. A systematic review found equivocal evidence regarding mortality differences between women and men with sepsis [13]. In contrast, a large prospective cohort of older adults reported higher risks of sepsis hospitalization, ICU admission, death, and rehospitalization among men [14]. More recently, sex-related differences in organ dysfunction severity at ICU admission, as measured by the Sequential Organ Failure Assessment (SOFA) score, were observed in patients with sepsis or SS, with more pronounced differences in younger patients, and a population-based cohort study suggested that sex-related outcome differences vary across the lifespan [15,16].

Taken together, these findings suggest that age and sex are not merely background characteristics, but clinically relevant determinants of sepsis phenotype and prognosis. Nevertheless, the interaction between age and sex remains insufficiently characterized, especially in prospective cohorts of patients with sepsis and SS. A better understanding of these associations may improve risk stratification and support more individualized management strategies. 

Therefore, the aim of the present study was to investigate the influence of age and sex on outcomes in patients with sepsis and SS using data from a prospective monocentric registry.

## 2. Materials and Methods

### 2.1. Patient Study, Design, and Data Collection

This study was designed as a registry-based prospective observational cohort study using data from the Mannheim Registry for Sepsis and Septic Shock (clinicaltrials.gov identifier: NCT05231720 (accessed on 14 March 2026)). The registry was conducted in accordance with the principles of the Declaration of Helsinki and approved by the Medical Ethics Committee II of the Medical Faculty Mannheim, University of Heidelberg, Germany (approval No. 2019-694N).

**Figure 1:**
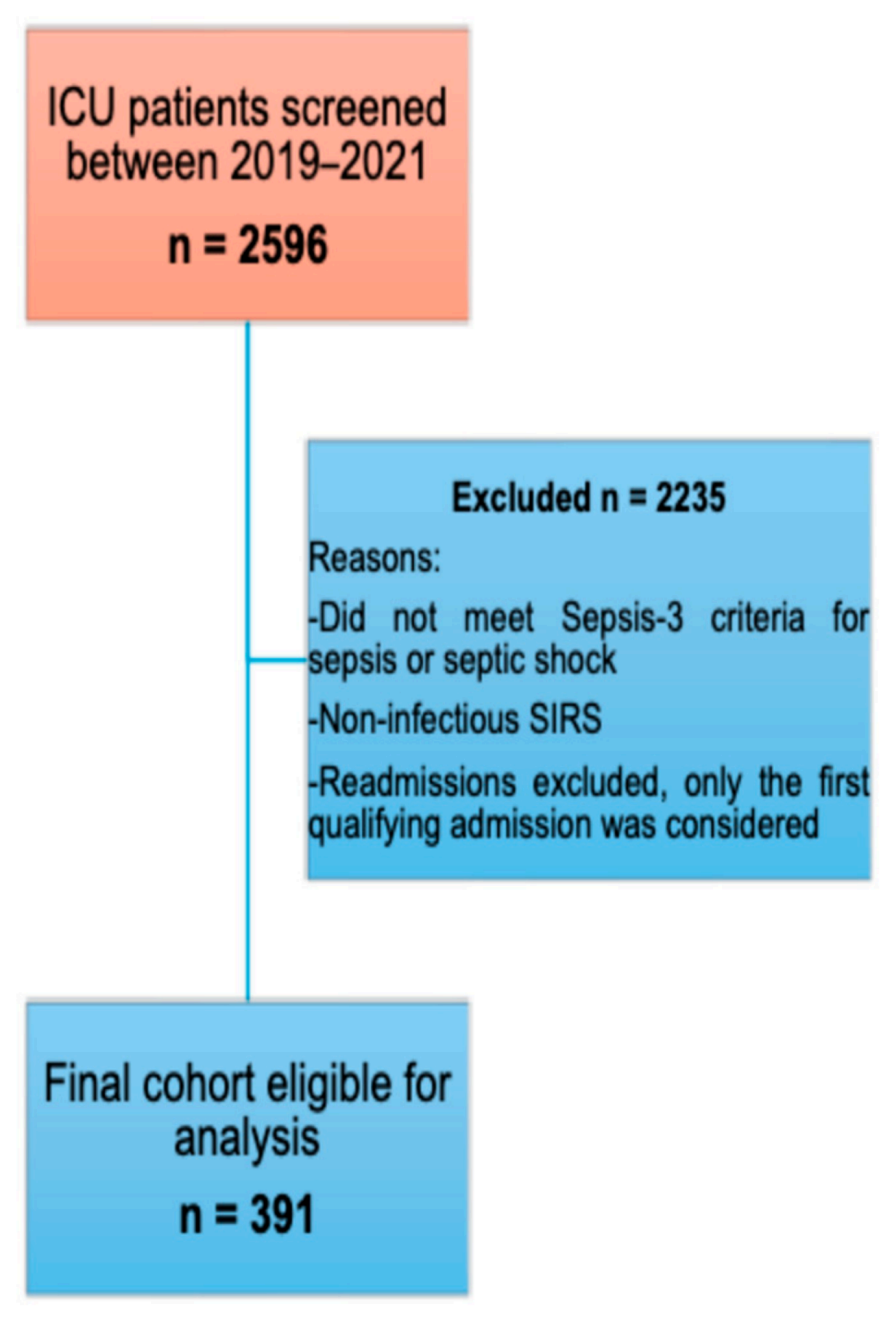
STROBR-style flow diagram of patient screening and inclusion.

Consecutive patients admitted to the ICU of the University Medical Center Mannheim, Germany, between 2019 and 2021 were screened for eligibility. During the study period, 2596 ICU patients were screened. Of these, 361 patients fulfilled Sepsis-3 criteria for sepsis/SS and were included in the final analytical cohort, as previously published [17,18]. The patient selection process is shown in the STROBE-style flow diagram in Figure 1.

All relevant clinical data related to the index event were extracted from the electronic hospital information system and the IntelliSpace Critical Care and Anesthesia information system (Philips GmbH Market DACH, Hamburg, Germany), which comprehensively document admission records, vital signs, laboratory values, treatment parameters, and consultation notes.

### 2.2. Inclusion and Exclusion Criteria, Risk Stratification, and Study Endpoints

Eligible patients were consecutive adult ICU patients who fulfilled the Sepsis-3 criteria for sepsis or SS during the study period. Patients with non-infectious systemic inflammatory response syndrome (SIRS) without suspected or confirmed infection and without Sepsis-3-defined organ dysfunction were not eligible. Readmissions were excluded, and only the first qualifying ICU admission was considered for analysis. Patients with documented treatment limitations, including do-not-resuscitate (DNR) orders, were included if they fulfilled Sepsis-3 criteria to reflect the “all-comers” ICU setting. No additional exclusion criteria were applied after fulfillment of the predefined eligibility criteria.

Diagnoses of sepsis and SS were made according to the Third International Consensus Definitions for Sepsis and Septic Shock (Sepsis-3) [1]. Sepsis was defined as life-threatening organ dysfunction caused by a dysregulated host response to infection, with organ dysfunction identified by an increase of ≥2 points in the SOFA score. SS was defined as persistent hypotension requiring vasopressors to maintain a mean arterial pressure ≥ 65 mmHg and lactate ≥2 mmol/L, despite adequate fluid resuscitation [1].

For the present study, risk stratification was performed according to sex (i.e., men and women), as well as according to age on hospital admission (≤75 vs. > 75 years, per 1-year increase).

The primary endpoint was all-cause mortality at 30 days. Mortality data were obtained through the hospital information system and verified via direct contact with state resident registration offices (Bureau of Mortality Statistics). No patient was lost to follow-up regarding 30-day all-cause mortality.

### 2.3. Statistical Methods

Continuous variables were presented as median values with interquartile ranges (IQRs) and were compared using the Student’s *t*-test for normally distributed data or the Mann–Whitney U test for non-normally distributed data, as appropriate. Normality was assessed using the Shapiro–Wilk test. Results of Shapiro–Wilk normality testing for selected continuous variables are provided in Supplementary Table S1. Categorical variables are presented as absolute and relative frequencies and were compared using the chi-square test or Fisher’s exact test, as appropriate.

Kaplan–Meier analyses were performed to evaluate 30-day survival in the overall cohort and in predefined subgroups according to age, sex, and disease severity. No patient was lost to follow-up for the primary endpoint.

Univariable and multivariable Cox proportional hazards regression analyses were performed to identify predictors of 30-day all-cause mortality. Variables included in the multivariable model were selected based on clinical relevance and potential confounding, including age, sex, diabetes mellitus, congestive heart failure, systolic blood pressure < 100 mmHg, malignancy, lactate > 2 mmol/L, sepsis versus septic shock, and mechanical ventilation at admission. To address the potential loss of information caused by age dichotomization, an additional sensitivity analysis was performed with age as a continuous variable. Interaction analyses were performed by adding age × sex interaction terms. Proportional hazards assumptions were assessed using supremum tests. In case of statistical significance, further analysis was applied to check the assumptions for the variables in the Cox regression model after 72 h of follow-up. All tests were two-sided, and *p* < 0.05 was considered statistically significant. Analyses were conducted using SPSS Statistics Version 28 (IBM, Armonk, NY, USA).

## 3. Results

### 3.1. Baseline Characteristics

A total of 361 patients with sepsis or SS were included, comprising 231 men and 130 women; 238 patients were aged ≤75 years and 123 were aged >75 years. Median age did not differ significantly between men and women, whereas women had a slightly higher body mass index (BMI) than men (27.55 vs. 26.23 kg/m^2^, *p* < 0.001). Baseline vital signs were comparable between sexes. Overall, baseline comorbidities were largely similar between men and women. In contrast, older patients had a lower BMI and a substantially higher burden of cardiovascular comorbidity, including arterial hypertension (79.7% vs. 56.5%, *p* < 0.001), coronary artery disease (CAD) (41.5% vs. 30.0%, *p* = 0.029), congestive heart failure (CHF) (26.8% vs. 15.6%, *p* = 0.011), atrial fibrillation (AF) (44.7% vs. 18.9%, *p* < 0.001), and chronic kidney disease (CKD) (31.7% vs. 13.4%, *p* < 0.001). By contrast, smoking and liver cirrhosis were more frequent in younger patients. Admission vital signs and left ventricular ejection fraction (LVEF) categories did not differ significantly between sex or age groups (Table 1).

### 3.2. Sepsis Characteristics and ICU Course

Regarding sepsis-related characteristics, the pulmonary tract was the most common source of infection in all subgroups, and the distribution of infection sources did not differ significantly by sex or age. Median time from hospital admission to ICU admission was also similar between groups. Severity of illness at presentation was comparable between men and women, with no significant differences in DIC, acute physiology, APACHE II, or SOFA scores. Across age groups, DIC, acute physiology, and SOFA scores were also similar; however, older patients had higher APACHE II scores (25 vs. 23, *p* = 0.001) and higher ISARIC-4C mortality scores (16 vs. 13, *p* < 0.001). SARS-CoV-2 infection and rates of cardiopulmonary resuscitation (CPR) were not significantly different between sex or age groups (Table 2).

Sex-related differences were mainly observed in treatment intensity and selected organ dysfunction markers. Dialysis during hospitalization was more frequent in men than in women (47.6% vs. 36.2%, *p* = 0.035). Acute liver failure occurred more often in men (10.8% vs. 4.6%, *p* = 0.043). Among laboratory parameters, men showed higher D-dimer and bilirubin levels, whereas women had higher troponin I levels; C-reactive protein (CRP) was also slightly higher in women (Table 2).

Age-related differences were more pronounced. Older patients had lower glomerular filtration rates (26.3 vs. 39.74 mL/min, *p* < 0.001). Thoracic CT imaging was performed less frequently in older patients (88.6% vs. 95.0%, *p* = 0.027). Younger patients had more blood cultures taken (median 5 vs. 3, *p* = 0.003), received higher norepinephrine doses (68.65 vs. 20.5 mg/mL, *p* = 0.009), and had longer durations of mechanical ventilation (8 vs. 3 days, *p* = 0.001) and ICU stay (10 vs. 5 days, *p* = 0.004) and longer dialysis duration (*p* = 0.015) (Table 2).

### 3.3. Outcomes and Survival Analyses

Thirty-day all-cause mortality did not differ between men and women (51.9% vs. 52.3%, *p* = 0.948), and ICU mortality was similar (50.2% vs. 46.2%, *p* = 0.459). In contrast, patients aged >75 years had higher 30-day mortality than those aged ≤75 years (60.2% vs. 47.9%, *p* = 0.027) (Table 2).

**Figure 2:**
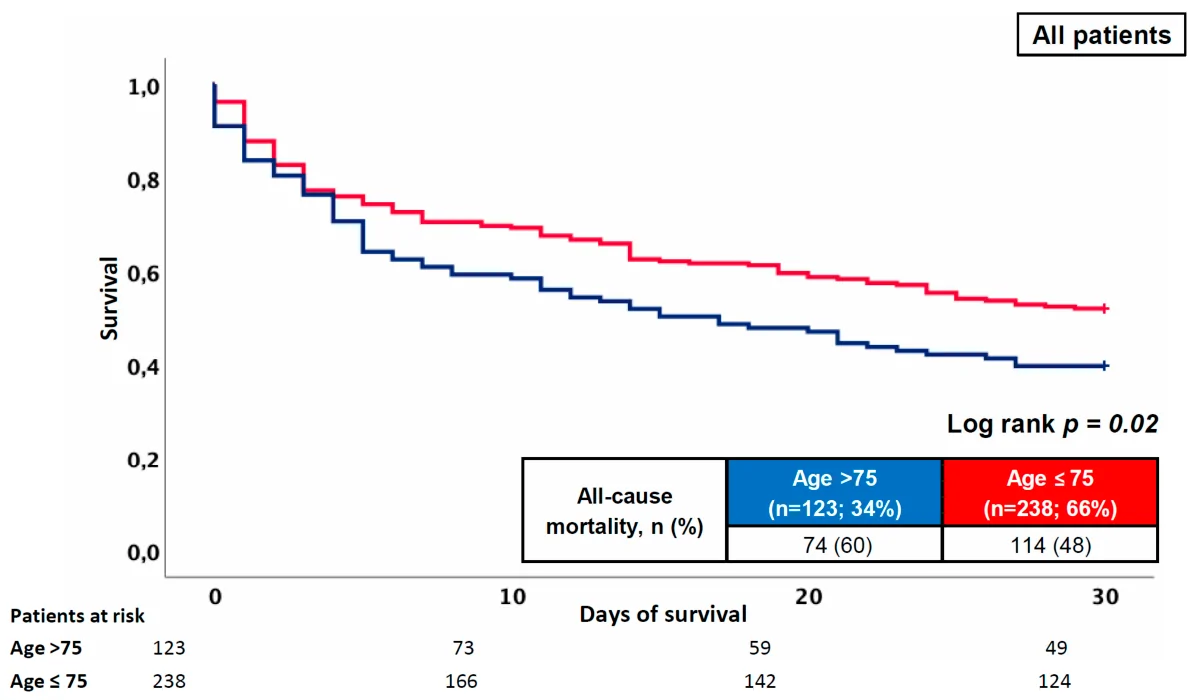
Prognostic impact of age on the risk of all-cause mortality at 30 days within the entire study cohort.

**Figure 3:**
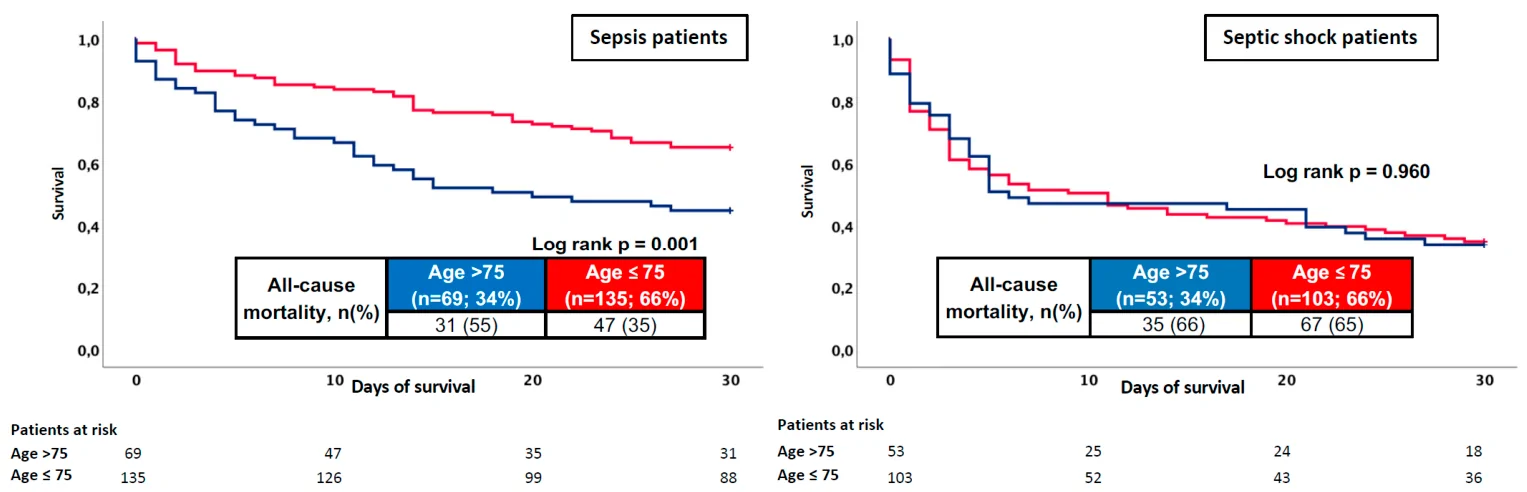
Prognostic impact of age on the risk of all-cause mortality at 30 days within sepsis patients (**left panel**) and patients with septic shock (**right panel**).

**Figure 4:**
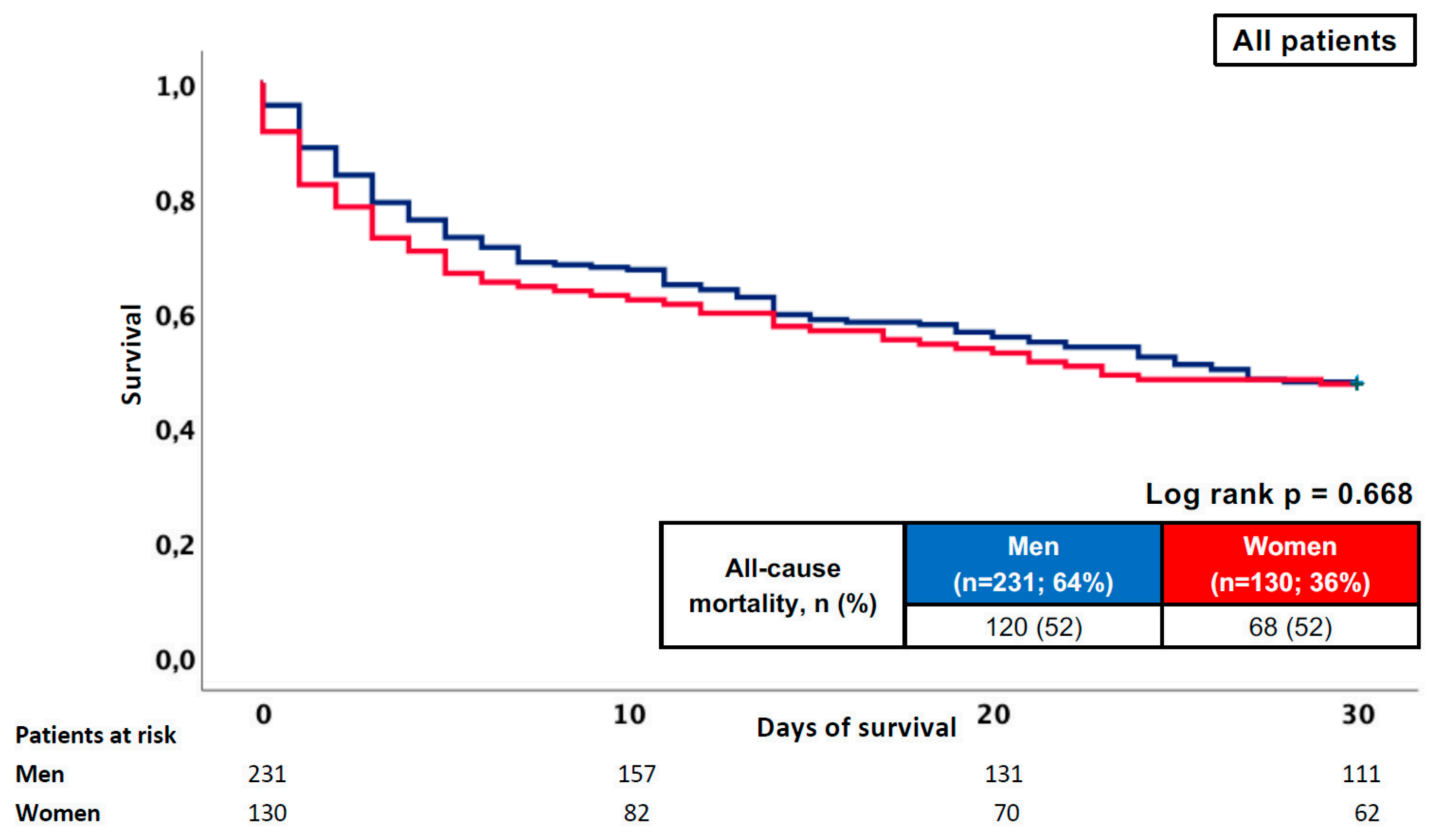
Prognostic impact of sex on the risk of all-cause mortality at 30 days within the entire study cohort.

**Figure 5:**
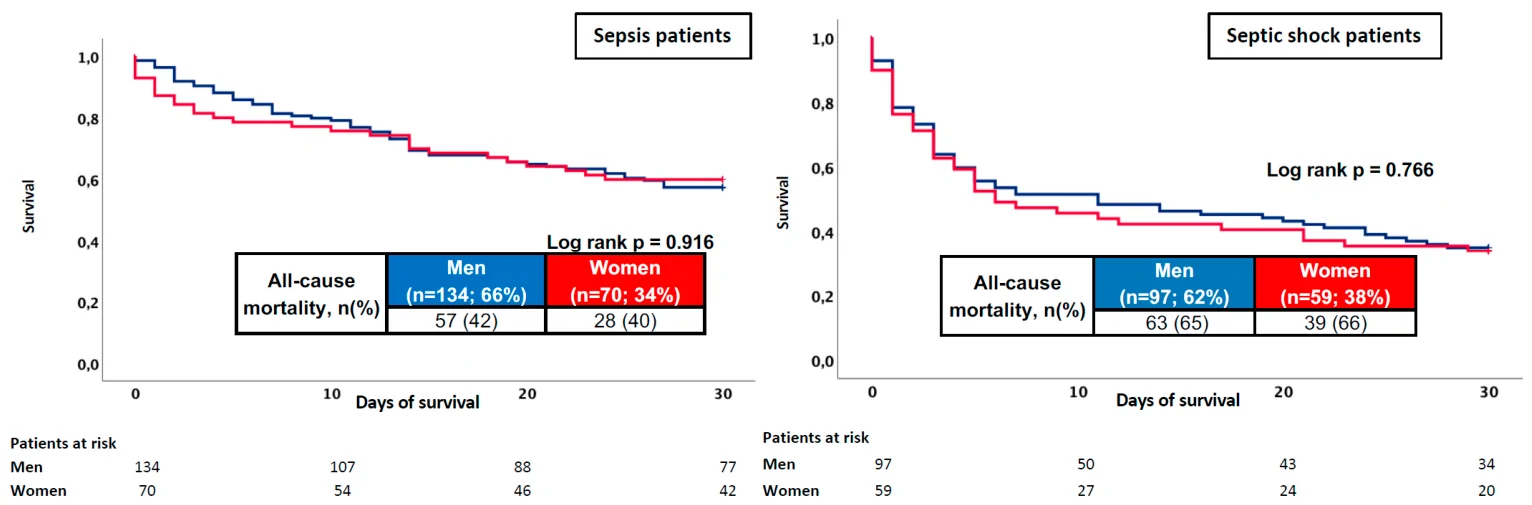
Prognostic impact of sex on the risk of all-cause mortality at 30 days within sepsis patients (**left panel**) and patients with septic shock (**right panel**).

In the overall cohort, survival differed significantly between age groups (log-rank *p* = 0.02), whereas no significant difference was observed between men and women (log-rank *p* = 0.668). In subgroup analyses stratified by disease severity, survival did not differ by age among patients with SS (log-rank *p* = 0.960), but a significant age-related difference was observed among patients with sepsis (log-rank *p* = 0.001). No significant sex-related survival differences were found either in patients with sepsis (log-rank *p* = 0.916) or in those with SS (log-rank *p* = 0.766) (Figure 2, Figure 3, Figure 4 and Figure 5).

### 3.4. Predictors of 30-Day Mortality

In the multivariable Cox regression model, age > 75 years remained independently associated with increased 30-day all-cause mortality (HR 1.391, 95% CI 1.021–1.896; *p* = 0.036). Lactate > 2 mmol/L was also independently associated with increased mortality (HR 1.585, 95% CI 1.112–2.261; *p* = 0.011). In contrast, sepsis compared with septic shock was associated with a lower hazard of death (HR 0.623, 95% CI 0.437–0.888; *p* = 0.009). Sex was not independently associated with 30-day mortality in the overall cohort (HR 1.016, 95% CI 0.740–1.396; *p* = 0.920). Diabetes mellitus, congestive heart failure, systolic blood pressure < 100 mmHg, malignancy, and mechanical ventilation at admission were not independently associated with mortality in the adjusted model (Table 3b).

To address the potential loss of information introduced by age dichotomization, age was additionally analyzed as a continuous variable. In univariable Cox regression, each one-year increase in age was associated with higher 30-day mortality (HR 1.013, 95% CI 1.002–1.024; *p* = 0.020) (Table 3a). This association remained significant after multivariable adjustment (HR 1.014, 95% CI 1.002–1.026; *p* = 0.023), confirming the robustness of the primary age-stratified analysis (Table 3b).

No significant interaction of age and sex was observed using the dichotomized age variable (*p* = 0.248) or age as a continuous variable (*p* = 0.424).

In subgroup Cox analyses, age > 75 years was independently associated with mortality among patients with sepsis (HR 2.286, 95% CI 1.439–3.632; *p* < 0.001), but not among patients with septic shock (HR 0.958, 95% CI 0.618–1.487; *p* = 0.850). Sex was not associated with mortality in either the sepsis subgroup (HR 0.928, 95% CI 0.562–1.533; *p* = 0.770) or the septic shock subgroup (HR 1.067, 95% CI 0.705–1.617; *p* = 0.758) (Table 4).

Assessment of the proportional hazard assumption indicated violations for selected covariates in the overall time-to-event analysis, including systolic blood pressure < 100 mmHg, lactate > 2 mmol/L, and sepsis vs. SS. Therefore, additional sensitivity checks were performed after the early 72 h phase. In these analyses, the proportional hazards assumption was no longer violated for systolic blood pressure < 100 mmHg, lactate > 2 mmol/L, or sepsis vs. SS, supporting the robustness of the overall Cox regression findings beyond the early acute phase (Supplementary Table S3). In patients with pneumogenic sepsis, age > 75 years remained associated with mortality (HR 1.632, 95% CI 1.100–2.422; *p* = 0.015), whereas this association was not observed in patients with non-pneumogenic sepsis (HR 1.181, 95% CI 0.704–1.982; *p* = 0.529). Conversely, lactate > 2 mmol/L and sepsis vs. SS were associated with outcome mainly in the non-pneumogenic subgroup. These subgroup analyses are shown in Supplementary Table S2 and should be considered exploratory.

## 4. Discussion

In this prospective, registry-based, single-center cohort of patients with sepsis and septic shock, the key findings were as follows: (1) older patients exhibited a higher burden of cardiovascular comorbidities and higher illness severity and had higher 30-day mortality; (2) age > 75 years remained independently associated with 30-day mortality in the updated multivariable Cox regression model; (3) sex was associated with differences in treatment intensity and selected markers of organ dysfunction, but was not independently associated with short-term mortality. Overall, these findings suggest that age and sex contribute differently to the heterogeneity of sepsis, with age more closely linked to short-term prognosis and sex more closely related to clinical phenotype and treatment exposure.

The observed 30-day mortality of approximately 50% to 60% is higher than that reported in some general sepsis cohorts [19,20]. This likely reflects the specific case mix of the present study, which included critically ill patients treated in a tertiary care ICU rather than all hospitalized patients with sepsis. The cohort had substantial illness severity, reflected by high APACHE II and SOFA scores, frequent vasopressor therapy, mechanical ventilation, and renal replacement therapy. In addition, the high burden of cardiovascular and renal comorbidities may have contributed to the elevated mortality. Therefore, the results should be interpreted in the context of a severely ill ICU population and may not be directly generalizable to lower-risk ward-based sepsis cohorts.

Population aging has markedly increased the proportion of individuals aged ≥60 years worldwide, from 8% in the 1950s to 10% in 2000, with projections of 21% by 2050 [9,21]. The population aged ≥80 years is also expected to double, reaching 9.6% in Europe and 9.0% in North America by 2050 [22]. This demographic shift is associated with a higher incidence and greater severity of sepsis, in both community- and hospital-acquired settings, among older adults than among younger individuals [23]. The resulting rise in sepsis-related incidence and mortality has created a substantial global health burden, prompting the World Health Organization (WHO) to recognize sepsis as a global health priority [24].

The association between older age and worse outcome is biologically plausible and consistent with the prior literature. Aging is accompanied by multimorbidity, frailty, reduced physiological reserve, and altered inflammatory responses, all of which may impair the ability to tolerate and recover from sepsis [9,10,25,26,27]. In the present cohort, older patients more frequently had hypertension, CAD, CHF, AF, and CKD and showed lower renal function and higher global severity scores. Notably, DIC, acute physiology, and SOFA scores did not differ significantly between age groups, whereas APACHE II and ISARIC-4C scores were higher in older patients. This pattern suggests that the adverse effect of age may not be fully explained by sepsis-related organ dysfunction at presentation alone but rather by broader host vulnerability and cumulative disease burden. This interpretation is in line with previous reports showing worse outcomes and more complex clinical trajectories in older, critically ill patients with sepsis [9,10,11].

The survival analyses further support the prognostic relevance of age. Overall survival was lower in patients aged >75 years, and age remained independently associated with death after multivariable adjustment. The robustness of the association between age and mortality was supported by sensitivity analyses. When age was modeled as a continuous variable, each one-year increase in age remained independently associated with 30-day mortality after adjustment. This reduces the concern that the observed association was solely driven by the selected age threshold. Nevertheless, dichotomization at 75 years was retained for the primary clinical presentation because it provides a pragmatic and clinically interpretable distinction between older and very old ICU patients. Subgroup Kaplan–Meier and Cox analyses suggested that the prognostic impact of age may vary according to disease severity. Age > 75 years was associated with mortality mainly among patients with sepsis, but not among those with septic shock. One possible explanation is that once severe circulatory and metabolic failure is established, the overwhelming physiological insult of septic shock may attenuate the relative contribution of chronological age, whereas in less extreme presentations, age-related differences in physiological reserve, comorbidity burden, and treatment tolerance remain more discernible. Although this interpretation remains exploratory, our data support the inclusion of age as an important component of risk stratification in patients with sepsis and SS [10,11,27,28]. Observational data from the ICON audit—comprising 10,069 patients from 730 centers across 84 countries—further support these findings by demonstrating that age > 70 years is independently associated with an increased risk of death, whereas age > 80 years confers the highest odds of death (OR 1.69, 95% CI 1.31–2.18) among ICU patients with sepsis worldwide [26].

In contrast, sex-related differences in this cohort were more apparent in treatment intensity and organ dysfunction patterns than in hard outcomes. Men had higher norepinephrine requirements, more frequent dialysis and a higher rate of acute liver failure. These findings are consistent with an emerging body of literature suggesting that sex influences both the host response to infection and the in-hospital management of sepsis [8,12,29]. Experimental and translational work has linked sex hormones and sex-chromosome-associated immune regulation to differences in innate and adaptive immune responses, endothelial dysfunction, and organ injury, although the clinical expression of these mechanisms is likely modified by age, comorbidity, and healthcare processes [12]. Recent clinical studies have likewise reported sex-related differences in bundle adherence, management patterns, and organ dysfunction severity in sepsis [8,16].

Despite these differences, sex was not associated with 30-day mortality in the present study. ICU mortality, 30-day mortality, Kaplan–Meier survival, and Cox regression analyses all yielded neutral findings regarding sex. Large cohort studies have reported higher rates of sepsis-related hospitalization, ICU admission, and death in men, while recent nationwide analyses suggest that sex-associated outcome differences may vary across the lifespan and may not be uniform across age strata or endpoints [14,15]. Our findings therefore support the view that sex remains a relevant source of heterogeneity in sepsis but may not exert a stable independent effect on short-term mortality in all ICU cohorts.

The interpretation of the neutral sex findings was refined by additional interaction analysis. No significant age × sex interaction was observed, either when age was analyzed dichotomously or continuously. Thus, within the limits of the present cohort, there was no evidence that the prognostic association of age differed significantly between men and women. However, this does not exclude smaller, sex-specific effects or age-dependent biological differences that may require larger multicenter cohorts to detect.

Another clinically relevant finding was the independent association of liver failure and LVEF with mortality. Although these variables were not the primary focus of the present study, they underscore that prognosis in sepsis is shaped by a combination of acute organ dysfunction, cardiovascular reserve, and host-related vulnerability. Reduced LVEF frequently reflects pre-existing cardiac disease in sepsis/SS, where sepsis-induced cardiomyopathy and chronic heart failure may coexist [30,31]. Similarly, the liver plays a central role in sepsis by contributing to bacterial clearance, acute-phase and cytokine responses, and metabolic adaptation to systemic inflammation. At the same time, it is highly susceptible to sepsis-related injury, including hypoxic hepatitis, cholestasis, drug- or inflammation-related hepatocellular damage, and secondary sclerosing cholangitis in critically ill patients. Accordingly, hepatic dysfunction is associated with impaired prognosis and represents an independent predictor of ICU mortality in sepsis [32,33]. Importantly, the persistence of age as an independent predictor after adjustment suggests that age acts as more than a surrogate for isolated organ impairment. Rather, chronological age likely integrates several unmeasured factors, including frailty, prehospital functional status, therapeutic limitations, and recovery potential, which are particularly relevant in critically ill older adults [10,25].

From a clinical perspective, these findings argue for a differentiated interpretation of age and sex in sepsis. Age should be regarded as an important prognostic marker that complements established severity scores and may help identify patients at increased risk of early death. In contrast, the observed sex-related differences may be more relevant for understanding variation in presentation, monitoring needs, and treatment exposure than for predicting short-term survival. These observations do not support sex-specific treatment recommendations based on this study alone, but they do support continued investigation into individualized approaches that integrate biological sex, age, comorbidity burden, and frailty [8,12,15,34,35].

### 4.1. Clinical Implications

In summary, in critically ill patients with sepsis or SS, older age was independently associated with higher 30-day mortality, while sex was not independently associated with mortality after adjustment. These findings support the integration of age-related vulnerability into early risk stratification and suggest that sex-related differences may be more relevant to clinical phenotype and treatment exposure than to short-term survival prediction in this cohort.

### 4.2. Study Strengths, Limitations, and Future Research

This study has several strengths. It was based on a prospective, well-characterized registry of patients with sepsis and SS defined according to Sepsis-3 criteria, included detailed ICU clinical and treatment data, and achieved complete 30-day follow-up. Several limitations should be acknowledged. First, the single-center observational design limits generalizability and precludes causal inference. Second, although 2596 ICU patients were screened, the final analytical cohort comprised 361 patients fulfilling Sepsis-3 criteria, which may limit statistical power for detecting modest sex-related effects or interaction effects. Third, age was primarily dichotomized at 75 years for clinical interpretability, although sensitivity analysis using age as a continuous variable confirmed the robustness of the association. Fourth, patients with treatment limitations, including DNR orders, were not excluded if they fulfilled Sepsis-3 criteria; however, treatment-limitation decisions were not included as covariates and may have contributed to residual confounding. Fifth, frailty scores, preadmission functional status, timing and adequacy of source control, and exact time-to-antibiotics were not systematically available. These variables are clinically relevant and may influence mortality in sepsis and septic shock. Finally, subgroup Cox analyses should be interpreted cautiously and considered as hypothesis-generating. Future multicenter studies should combine detailed geriatric assessment with sex-specific biological and treatment-related variables to better clarify how age and sex interact in shaping sepsis phenotype and outcome.

## Figures and Tables

**Table 1:** Baseline characteristics of the study population.

Characteristic	Male (*n* = 231)		Female (*n* = 130)		*p* Value	Age ≤ 75 (*n* = 238)		Age > 75 (*n* = 123)		*p* Value
**Age**, median (IQR)	70	60–78	69	59–81	0.332	63	55–69	81	79–84	**<0.001**
**Male sex**, *n* (%)	231	(100)	0	(0)	**<0.001**	157	(66.0)	74	(60.2)	0.276
**Body mass index** (kg/m^2^, median (IQR))	26.23	23.4–29.4	27.55	23.4–33.1	**<0.001**	26.63	23.44–30.86	25.95	23.32–29.38	**0.018**
**Entry criteria**, median (IQR)										
Body temperature (°C)	36.6	35.9–37.5	36.8	36–37.5	0.567	36.7	36–37.6	36.7	35.8–37.3	0.494
Heart rate, bpm	95	85–112	104	86–120	0.081	99	86–113	96	84–117	0.292
Systolic BP, mmHg	112	97–131	106	89–126	0.927	108	94–126	116	95.75–132.25	0.213
Respiratory rate, breaths/min	22	18–26	20	17–27	0.237	21	18–26	22	17–27.25	0.836
**Cardiovascular risk factors**, *n* (%)										
Arterial hypertension	150	(65.2)	82	(63.1)	0.684	134	(56.5)	98	(79.7)	**<0.001**
Diabetes mellitus	70	(30.4)	51	(39.2)	0.090	72	(30.4)	49	(39.8)	0.072
Hyperlipidemia	61	(26.6)	42	(32.3)	0.254	61	(25.8)	42	(34.1)	0.099
Smoking	68	(29.8)	36	(27.9)	0.702	83	(35.5)	21	(17.1)	**<0.001**
**Prior medical history**, *n* (%)										
Coronary artery disease	85	(37.0)	37	(28.5)	0.102	71	(30.0)	51	(41.5)	**0.029**
Congestive heart failure	44	(19.1)	26	(20.0)	0.841	37	(15.6)	33	(26.8)	**0.011**
Atrial fibrillation	60	(26.0)	40	(30.8)	0.328	45	(18.9)	55	(44.7)	**<0.001**
Chronic kidney disease	43	(18.6)	28	(21.5)	0.502	32	(13.4)	39	(31.7)	**<0.001**
COPD	43	(18.7)	26	(20.0)	0.763	50	(21.1)	19	(15.4)	0.196
Liver cirrhosis	27	(11.7)	8	(6.2)	0.088	32	(13.4)	3	(2.4)	**0.001**
Malignancy	77	(33.3)	40	(30.8)	0.617	74	(31.1)	43	(35.0)	0.457
Immunosuppression	36	(16.1)	16	(12.8)	0.401	38	(16.5)	14	(11.9)	0.249
**LVEF index at admission**, *n* (%)					0.537					
≥55%	90	(42.5)	49	(40.5)		102	(45.5)	37	(33.9)	0.078
54–45	52	(24.5)	38	(31.4)		58	(25.9)	32	(29.4)	
44–35%	38	(17.9)	17	(14.0)		30	(13.4)	25	(22.9)	
<35%	32	(15.1)	17	(14.0)		34	(15.2)	15	(13.8)	

COPD, chronic obstructive pulmonary disease; IQR, interquartile range; LVEF, left ventricular ejection fraction; Systolic BP, systolic blood pressure. Level of significance *p* < 0.05.

**Table 2:** Sepsis-related data, follow-up data and endpoints.

Characteristic	Male (*n* = 231)		Female (*n* = 130)		*p* Value	Age ≤ 75 (*n* = 238)		Age > 75 (*n* = 123)		*p* Value
**Median days in hospital before ICU admission**, median (IQR)	0	(0–5)	0	(0–4)	0.601	0	(0–5)	0	(0–3)	0.549
**Sepsis scores**, median (IQR)										
DIC	1	0–2	1	0–2	0.707	1	0–2	1	0–2	0.307
Acute physiology score	15	11–21	17	12–21	0.151	16	11–21.25	17	12–21	0.236
APACHE II	23	17–29	24	18–29	0.185	23	17–28	25	19–30	**0.001**
SOFA	11	8–14	10	7.75–13	0.307	11	8–14	11	8–13	0.393
ISARIC-4C-Mortality Score	15	12–16	13	11–15	0.741	13	10–15	16	15–17	**<0.001**
**Source of Sepsis**, *n* (%)					0.953					0.575
Lung	143	(58.6)	71	(60.7)		136	(57.1)	78	(63.4)	
Urogenital	28	(11.5)	10	(8.5)		25	(10.5)	13	(10.6)	
Intra-abdominal	23	(9.4)	10	(8.5)		25	(10.5)	8	(6.5)	
Wound	1	(0.4)	1	(0.9)		1	(0.4)	1	(0.8)	
Unknown	47	(19.3)	24	(20.5)		48	(20.2)	32	(18.7)	
SARS-CoV-2 infection, *n* (%)	28	(12.1)	14	(10.8)	0.701	28	(11.8)	14	(11.4)	0.914
**Cardiopulmonary resuscitation**, *n* (%)	24	(10.4)	18	(13.8)	0.325	28	(11.8)	14	(11.4)	0.914
In-hospital	6	(2.6)	7	(5.4)	0.377	8	(3.4)	5	(4.1)	0.890
Out of hospital	18	(7.8)	11	(8.5)		20	(8.4)	9	(7.3)	
**Antibiotic treatment at index**, *n* (%)										
Beta-lactam	187	(81.0)	112	(86.2)	0.208	192	(80.7)	107	(87.0)	0.131
Vancomycin	14	(16.1)	5	(3.8)	0.366	15	(6.3)	4	(3.3)	0.219
Macrolide	10	(4.3)	8	(6.2)	0.444	13	(5.5)	5	(4.1)	0.563
Linezolid	9	(3.9)	1	(0.8)	0.082	7	(2.9)	3	(2.4)	0.783
**Microbiology**, median (IQR)										
Numbers of blood cultures taken	4	2–8	3	2–6	**0.026**	5	2–9	3	2–5	**0.003**
Positive blood culture	0	0–2	0	0–2	0.148	0	0–2	0	0–1	0.200
**Fluid balance at admission to ICU** (mL/24 h; median (IQR))	10,683	4214.3–18,770.5	7883	3157.8–15,788.5	**0.014**	1811	340–4282	1839	704–3953.5	0.924
**Multiple organ support during ICU**										
Vasopressor support norepinephrine, *n* (%)	204	(88.3)	111	(85.4)	0.423	211	(88.7)	104	(84.6)	0.268
Dosis norepinephrine (µg/kg/min; median (IQR))	0.10	0.00—0.40	0.10	0.09—0.30	**0.002**	0.10	0.045—0.50	0.10	0.00—0.20	**0.009**
Dialysis during hospitalization, *n* (%)	110	(47.6)	47	(36.2)	**0.035**	110	(46.2)	47	(38.2)	**0.002**
Extracorporeal membrane oxygenation, *n* (%)	19	(8.2)	5	(3.8)	0.109	122	(99.2)	1	(0.8)	
**Respiratory status**										
Mechanical ventilation (MV), *n* (%)	121	(52.4)	72	(55.4)	0.583	127	(53.4)	66	(53.7)	0.957
Invasive mechanical ventilation, *n* (%)	92	(39.8)	58	(44.6)	0.375	105	(44.1)	45	(36.6)	0.169
Duration of MV (days; mean (range))	6	1–17	5	1–14	0.107	8	2–17	3	0–9	**0.001**
**Central nervous system**										
GCS (mean (range))	6	3–13	3	3–12	0.160	3	3–13	7.5	3–13	0.969
Delirium, *n* (%)	27	(11.7)	17	(13.1)	0.699	25	(10.5)	19	(15.4)	0.174
**Liver function**										
Acute liver failure, *n* (%)	25	(10.8)	6	(4.6)	**0.043**	23	(9.7)	8	(6.5)	0.310
**Renal function**, median (IQR)										
Creatinine (mg/dL)	1.91	1.16–3.07	1.63	1.05–2.55	0.343	1.63	0.99–2.74	2.26	1.37–3.17	0.193
GFR (mL/min)	35.22	20.12–61.12	31.32	18.07–53.75	0.456	39.74	22.14–69.76	26.3	16.65–41.99	**<0.001**
Urine output (mL)	825	230–1587.5	750	202.5–1530	0.946	865	247.5–1642.5	670	197.5–1412.5	0.116
Dialysis (days)	0	0–4	0	0–2.25	0.206	0	0–5	0	0–2	**0.015**
**Baseline laboratory values**, median (IQR)										
pH	7.37	7.28–7.43	7.37	7.29–7.42	0.209	7.37	7.28–7.42	7.36	7.29–7.43	0.475
BE (mmol/L)	−2	−6.3–1.1	−2.8	−7.05–2.85	0.191	−1.7	−5.95–2.05	−3.2	−8.18–0.6	0.020
Lactate (mmol/L)	2	1.275–4.1	1.9	1.2–3.95	0.946	2	1.2–3.9	2.1	1.3–4.5	0.741
Sodium (mmol/L)	139	134–144	140	136–146	0.804	139	134–145	140	136.25–144	0.179
Potassium (mmol/L)	4.2	3.9–4.8	4.1	3.7–4.5	0.335	4.2	3.78–4.8	4.1	3.8–4.55	0.263
Hemoglobin (g/dL)	9.9	8.2–12.3	9.85	8.48–11.6	0.353	9.7	8.13–12.1	10.5	8.6–12.3	0.390
WBC (10^9^ cells/mL)	12.13	7.95–17.67	13.65	8.61–20.94	0.727	12.29	7.86–17.07	13.69	8.38–21.64	0.198
Platelets (10^9^ cells/mL)	164	101.25–261.75	197	123.75–270	0.344	168	101–268	192	130–267	0.702
INR	1.19	1.09–1.37	1.2	1.07–1.39	0.176	1.2	1.07–1.39	1.19	1.1–1.33	0.102
Fibrinogen (g)	3.4	2.5–5.43	3.87	2.58–5.92	0.079	3.95	2.4–5.76	3.31	2.6–5.1	0.502
D-dimer (µg/L)	5.22	1.81–19.42	4.13	1.42–12.57	**0.046**	4.13	1.33–19.67	6.33	2.07–13.74	0.523
ASAT	56.5	29–127	57	30–127.25	0.607	60	31–130	47	26.5–107	0.822
ALAT	31	17–76	31	18.25–71.5	0.136	33	19–78.25	25	17–62	0.757
Bilirubin (mg/dL)	0.96	0.55–1.82	0.73	0.45–1.61	**0.009**	0.97	0.52–1.98	0.76	0.49–1.24	**0.007**
Troponin I (µg/L)	0.14	0.04–0.91	0.33	0.05–1.05	**0.007**	0.28	0.03–0.97	0.21	0.06–0.97	0.369
BNP (pg/mL)	2660	789–7835	2974.5	1213.5–8121	0.446	2451.5	623–7977.5	3033	1503.5–7871	0.704
Procalcitonine (ng/mL)	3.08	0.77–18.4	2.18	0.68–23.3	0.318	2.32	0.79–14.4	2.8	0.6–39.02	0.261
CRP (mg/dL)	143	87.2–213	147	78–231	**0.030**	145	81.93–218.5	144	89.35–220.5	0.958
**Primary endpoint**										
All-cause mortality at 30 days, *n* (%)	120	(51.9)	68	(52.3)	0.948	114	(47.9)	74	(60.2)	**0.027**
**Follow-up data**, *n* (%)										
ICU time (days; median (IQR))	8	3–20	6.5	3–16.25	0.265	10	4–22	5	2–13	**0.004**
Death ICU, *n* (%)	116	(50.2)	60	(46.2)	0.459	118	(49.6)	58	(47.2)	0.662

APACHE II, acute physiology and chronic health evaluation II; BE, base excess; BNP, brain natriuretic peptide; CT, computer tomography; CRP, C-reactive Protein; DIC, disseminated intravascular coagulation; GCS, Glasgow Coma Scale; GFR, glomerular filtration rate; ICU, intensive care unit; INR, international normalized ratio; IQR, interquartile range; SOFA, sepsis-related organ failure assessment score; WBC, white blood cell. Level of significance *p* < 0.05.

**Table 3:** (**a**) Univariable Cox regression analyses in the overall cohort. (**b**) Multivariable Cox regression model in the overall cohort.

**(a)**			
**Variable**	**HR**	**95% CI**	***p* Value**
**Sex (female vs. male)**	1.066	0.788–1.430	0.675
**Diabetes mellitus**	0.925	0.678–1.250	0.618
**Congestive heart failure**	0.963	0.659–1.367	0.838
**Systolic BP < 100 mmHg**	0.863	0.560–1.300	0.493
**Malignancy**	1.206	0.894–1.627	0.221
**Lactate > 2 mmol/L**	2.030	1.499–2.749	**<0.001**
**Sepsis vs. septic shock**	0.658	0.448–0.973	**0.034**
**Mechanical ventilation at admission**	1.191	0.893–1.590	0.234
**Age > 75 years**	1.405	1.044–1.878	**0.023**
**Age, per 1-year increase**	1.013	1.002–1.024	**0.020**
**(b)**			
**Variable**	**HR**	**95% CI**	***p* Value**
**Diabetes mellitus**	0.947	0.676–1.326	0.750
**Congestive heart failure**	0.939	0.638–1.383	0.751
**Systolic BP < 100 mmHg**	0.964	0.694–1.340	0.827
**Malignancy**	1.083	0.780–1.502	0.634
**Lactate > 2 mmol/L**	1.585	1.112–2.261	**0.011**
**Sepsis vs. septic shock**	0.623	0.437–0.888	**0.009**
**Mechanical ventilation at admission**	1.123	0.825–1.527	0.462
**Age > 75 years**	1.391	1.021–1.896	**0.036**
**Sex (female vs. male)**	1.016	0.740–1.396	0.920
Age *, per 1-year increase	1.014	1.002–1.026	**0.023**

(a): HR, hazard ratio; CI, confidence interval; BP, blood pressure. Level of significance *p* < 0.05. (b): HR, hazard ratio; CI, confidence interval; systolic BP, systolic blood pressure. Level of significance *p* < 0.05. * Multivariable adjustment was performed for the same variables as in the original model.

**Table 4:** Multivariable Cox regression analyses stratified by sepsis and septic shock.

Variable	Sepsis HR (95% CI)	*p* Value	Septic Shock HR (95% CI)	*p* Value
**Diabetes mellitus**	0.802 (0.474–1.356)	0.410	1.148 (0.737–1.788)	0.541
**Congestive heart failure**	0.865 (0.466–1.605)	0.645	0.899 (0.542–1.491)	0.681
**Systolic BP < 100 mmHg**	0.824 (0.478–1.418)	0.484	1.061 (0.693–1.625)	0.784
**Malignancy**	1.443 (0.874–2.384)	0.152	0.955 (0.616–1.479)	0.836
**Lactate > 2 mmol/L**	1.560 (0.953–2.554)	**0.077**	1.735 (0.994–3.028)	**0.053**
**Mechanical ventilation at admission**	1.280 (0.808–2.027)	0.293	1.033 (0.679–1.571)	0.881
**Age > 75 years**	2.286 (1.439–3.632)	**<0.001**	0.958 (0.618–1.487)	0.850
**Sex (female vs. male)**	0.928 (0.562–1.533)	0.770	1.067 (0.705–1.617)	0.758

HR, hazard ratio; CI, confidence interval; BP, blood pressure. Level of significance *p* < 0.05.

## Data Availability

The data underlying this article will be shared upon reasonable request to the corresponding author.

## Supplementary Materials

The following supporting information can be downloaded at: https://www.mdpi.com/article/10.3390/jcm15114203/s1, Supplementary Table S1: Shapiro–Wilk normality testing for selected continuous variables; Supplementary Table S2: Subgroup Cox regression analyses according to source of infection; Supplementary Table S3: Proportional hazards assumption diagnostics.
