# Supplementary material for: Age- and Sex-Related Outcomes in Patients with Sepsis or Septic Shock: A Prospective Monocentric Cohort Study
Source: J Clin Med. 2026 May 29;15(11):4203. doi: 10.3390/jcm15114203 (PMC13257473; doi:10.3390/jcm15114203)
Supplement: Supplementary file 1 [file jcm-15-04203-s001.zip › jcm-4264578-supplementary.pdf]

Supplementary Tables

Supplementary Table S1. Shapiro–Wilk normality testing for selected continuous variables.

Variable	Group	n	Shapiro–Wilk W	p value
**Age**	Male	231	0.946	<0.001
	Female	130	0.966	0.002
**Age**	≤75 years	238	0.896	<0.001
	>75 years	123	0.932	<0.001
**BMI**	Male	224	0.898	<0.001
	Female	122	0.865	<0.001
**BMI**	≤75 years	232	0.864	<0.001
	>75 years	114	0.832	<0.001
**Temperature at admission**	Male	222	0.953	<0.001
	Female	125	0.946	<0.001
**Heart rate at admission**	Male	231	0.976	0.001
	Female	129	0.978	0.031
**Systolic BP at admission**	Male	231	0.966	<0.001
	Female	128	0.969	0.005
**Respiratory rate at admission**	Male	229	0.971	<0.001
	Female	129	0.963	0.001
**ICU length of stay**	Male	231	0.722	<0.001
	Female	130	0.622	<0.001

Supplementary Table S2. Subgroup Cox regression analyses according to source of infection.

Variable	Pulmonary source HR (95% CI)	p value	Non-pulmonary source HR (95% CI)	p value
**Diabetes mellitus**	1.137 (0.724-1.784)	0.577	0.798 (0.470-1.356)	0.404
**Congestive heart failure**	1.016 (0.621-1.664)	0.950	0.814 (0.428-1.548)	0.529
**Systolic BP <100 mmHg**	0.841 (0.542-1.306)	0.441	1.081 (0.647-1.807)	0.765
**Malignancy**	1.143 (0.745-1.753)	0.541	1.049 (0.625-1.763)	0.856
**Lactate >2 mmol/L**	1.436 (0.887-2.323)	0.141	1.756 (1.003-3.074)	**0.049**
**Sepsis vs. septic shock**	0.675 (0.416-1.098)	0.113	0.534 (0.312-0.914)	0.022
**Mechanical ventilation at admission**	0.986 (0.655-1.484)	0.947	1.362 (0.839-2.212)	0.212
**Age >75 years**	1.632 (1.100-2.422)	**0.015**	1.181 (0.704-1.982)	0.529
**Sex (female vs. male)**	0.926 (0.593-1.447)	0.736	1.074 (0.670-1.724)	0.766

Abbreviations: BP, blood pressure; CI, confidence interval; HR, hazard ratio.

Supplementary Table S3. Proportional hazards assumption diagnostics.

Variable	Supremum test p value
**Female sex vs. male sex**	0.428
**Diabetes mellitus**	0.615
**Congestive heart failure**	0.754
**Malignancy**	0.801
**Mechanical ventilation at admission**	0.672
**Systolic BP <100 mmHg**	0.135
**Lactate >2 mmol/L**	0.425
**Sepsis vs. septic shock**	0.101
**Age, per 1-year increase**	0.907

**Age >75 years**	0.369

Proportional hazards assumptions were assessed using supremum tests. Values >0.05 indicate no evidence of violation for the corresponding variable.
*p value after censoring patients with early death < 72 hours.
